# Comparative analysis reveals distinct metal and inflammatory cytokine profiles in the cerebrospinal fluid of children with neurological disorders

**DOI:** 10.3389/ftox.2026.1766904

**Published:** 2026-03-24

**Authors:** Debora Curci, Martina Franzin, Enrico Pobega, Stefania Braidotti, Anna Flamigni, Gilda Paternuosto, Giulia Schillani, Riccardo Addobbati, Stefania Norbedo, Natalia Maximova

**Affiliations:** 1 Advanced Translational Diagnostic Laboratory, Institute for Maternal and Child Health-IRCCS Burlo Garofolo, Trieste, Italy; 2 Department of Pediatrics, Institute for Maternal and Child Health-IRCCS Burlo Garofolo, Trieste, Italy; 3 Pharmacy and Clinical Pharmacology Department, Institute for Maternal and Child Health-IRCCS Burlo Garofolo, Trieste, Italy; 4 Emergency Department, Institute for Maternal and Child Health-IRCCS Burlo Garofolo, Trieste, Italy

**Keywords:** cerebrospinal fluid, cytokine, inflammation, metal, metalloid, pediatric

## Abstract

**Introduction:**

The adverse effect of metals and metalloids on neural development is a significant health concern, especially in vulnerable populations, such as pediatric patients. Excessive accumulation of these elements in the developing central nervous system (CNS) can induce oxidative stress, trigger cell death, and promote neuroinflammation. However, few studies have quantified metal and metalloid concentrations in cerebrospinal fluid (CSF) in pediatric patients, and, to our knowledge, none have compared these levels between children with and without neurological disorders.

**Methods:**

In this exploratory study, we compared pediatric patients with neurological diseases or CNS infections to control pediatric patients to assess differences in metal and metalloid concentrations and in CSF inflammatory cytokine profiles.

**Results:**

We observed higher levels of most metals and metalloids in neurological and infection patients, while group-specific differences were observed in cytokines. Notably, the cytokines Pentraxin-3 and IL-8 showed positive correlations with calcium, copper, iron, antimony, and chromium, suggesting a possible functional relationship in neuroinflammation.

**Discussion:**

In conclusion, this study presents a comparative analysis of CSF metal and metalloid levels in conjunction with inflammatory cytokine profiles in a pediatric population, providing a basis for further research into their roles in neuroinflammation and neurological disease.

## Introduction

Childhood neurological disorders are a public health challenge, often linked to unknown factors. In addition to genetics, exposure to metals and metalloids can disrupt neurodevelopment and cause neurotoxicity (da Silva et al., 2025), potentially leading to cognitive and behavioral problems. Metals are part of the Earth’s crust and are present in tiny amounts in water, air, and different ecosystems. They can typically be categorized into two groups: essential and non-essential metals. Essential metals are usually required in trace amounts to facilitate a wide range of physiological processes, including electron transport, oxygen transportation, protein modification, neurotransmitter synthesis, redox reactions, immune responses, cell adhesion, and protein and carbohydrate metabolism (Chen et al., 2016). To shield the central nervous system (CNS) from potentially harmful molecules, the human organism employs semipermeable barriers such as the blood–brain barrier (BBB) and blood-cerebrospinal fluid barrier (BCB). In fact, in the capillaries of the CNS microvasculature, the wedged endothelial cells line the interior vessels, forming extensive tight junctions (Wu D. et al., 2023). Numerous inorganic elements, including iron, copper, zinc, calcium, manganese, and selenium, are nonetheless transferred by metal transporters to the brain, as required for regular neuronal activity (Takeda, 2004; Grochowski et al., 2019). Metal transporters lack high levels of specificity, a feature that facilitates the efficient transport of multiple cargos. Conversely, there is a mechanism known as molecular mimicry, which allows potentially toxic molecules with similar physical or chemical properties to essential elements to be delivered into the brain (Caito and Aschner, 2015). Elevated metal levels can disrupt neurotransmission and lead to neurodegeneration, resulting in cognitive impairments, including learning and memory problems, and movement disorders (Wright and Baccarelli, 2007; Shaw and Tomljenovic, 2013; Zhang et al., 2013). To date, metal-induced neurotoxicity has been linked to various neurological diseases in humans, including Alzheimer’s disease, amyotrophic lateral sclerosis, Guillain–Barré syndrome, Gulf War syndrome, Huntington’s disease, manganism, multiple sclerosis, Parkinson’s disease, Wilson’s disease, attention deficit hyperactivity disorder (ADHD), and autism spectrum disorders (ASD) (Strong et al., 1996; Okuda et al., 1997; Authier et al., 2001; Strausak et al., 2001; Desai and Kaler, 2008; Chen et al., 2015; da Silva et al., 2025). The health effects on the pediatric population are particularly relevant, as the developing human brain is uniquely vulnerable to exposure to metals and metalloids. The main windows of developmental vulnerability occur *in utero* and during infancy and early childhood (Rice and Barone, 2000). During these susceptible life stages, chemicals can cause permanent brain damage at low levels of exposure, which would have little or negligible harmful effect on an adult (Grandjean and Landrigan, 2014). Worldwide, serious concern has arisen over the increasing rates of learning and neurobehavioral disorders in children (ADHD, ASD, anxiety), and their potential link to exposure to neurotoxic chemicals during early brain development. Research conducted in the 1960s in Europe and the US suggested that ASD was an uncommon condition, with an incidence of two to four children per 10,000 (Sauer et al., 2021). Recent research shows that one in six children in the United States has ADHD, oppositional defiant disorder, ASD, anxiety disorder, learning disorders, or conduct disorders (CDC, 2025). Substantial evidence indicates that industrial chemicals widely present in the environment are major contributors to the so-called global silent pandemic of neurodevelopmental toxicity (Grandjean and Landrigan, 2006).

Neuroinflammation is a key mechanism underlying CNS diseases and plays a particularly important role during brain development, when the CNS is highly vulnerable to environmental insults (Wei et al., 2024). Among these insults, metals have been strongly implicated in pediatric neurodevelopmental impairment, in part through inflammation-mediated pathways (Wei et al., 2024; da Silva et al., 2025). For example, co-exposure to lead and manganese during childhood has been associated with long-lasting neurodevelopmental consequences, including intellectual dysfunction and behavioral abnormalities (Kim et al., 2009). Lead exposure in children has also been linked to an increased risk of attention-deficit/hyperactivity disorder (ADHD), potentially mediated by alterations in synapse density, neuronal activity, neurotransmission, and calcium signaling (Desye et al., 2023; Rosenauer et al., 2024). In addition, prenatal exposure to cadmium, arsenic, mercury, manganese, and lead has been associated with postnatal cognitive deficits, including impairments in executive function, verbal abilities, motor skills, and quantitative reasoning (Freire et al., 2018).

A steady rise in neurobehavioral disorders, and most notably, a decrease in the age at which the first symptoms appear, has been observed in the clinical setting of our pediatric hospital and worldwide (Feigin et al., 2020). However, conducting an exploratory study of CNS exposure to toxic metals in a local pediatric cohort is challenging due to the near-complete lack of reference values for metal levels in children’s CSF. As a result, many studies rely on blood measurements as a surrogate (Goullé et al., 2005; Heitland and Köster, 2006; Bocca et al., 2010). However, blood levels do not accurately reflect CNS exposure, especially given that the BBB may exhibit varying permeability depending on the patient’s age and underlying morbidities.

The primary aim of our study is to provide an overall estimate of the levels of metals and metalloids in pediatric CSF and to assess differences between patients with and without CNS diseases. In parallel, we evaluated inflammatory cytokine profiles to explore associations between metal and metalloid levels and neuroinflammatory status. To our knowledge, few studies have quantified metals and metalloids in the CSF of pediatric patients (Tondo et al., 2010; Franěk et al., 2017; Wu W. et al., 2023) and none have concomitantly assessed these concentrations alongside inflammatory molecules. Understanding the connection between metal levels and neuroinflammatory status, as assessed by cytokine profiling, is essential for identifying potential biomarkers and developing therapeutic strategies.

## Materials and methods

### Study design

A single-center, observational, retrospective study was conducted at the Institute for Maternal and Child Health - IRCCS Burlo Garofolo, in Trieste, Italy, following approval from the local Institutional Review Board (reference No. IRB RC 15/25). The study was conducted in accordance with the principles outlined in the Declaration of Helsinki.

### Study population and sample collection

From 2014 to 2024, 157 patients aged 0–17 years were retrospectively enrolled. Patients were included in the study if their parents or legal guardians provided informed consent for the utilization of the discarded biological samples for research purposes. The CSFs of untreated oncological patients assisted during the years 2014–2024 in the Pediatric Oncology and Hematology Department were included in the Control Group (n = 69). To ensure that the CSF samples could serve as controls, children with CNS leukemia were excluded from the study. The CSFs of patients who accessed the Emergency Department during the same years and were diagnosed with neuropsychiatric disease complications or CNS infections were included as the Case Group (n = 88).

All patients underwent a lumbar puncture for diagnostic purposes. Amounts of CSF remaining after laboratory tests were collected into sterile tubes. All samples were centrifuged immediately after collection (4000 RCF, 10 min, +4 °C) to remove cellular debris, then stored at −80 °C. Before analysis, the samples were thawed, briefly mixed, and then centrifuged again to remove any remaining debris. Patients whose legal guardians did not provide informed consent were excluded from the study.

### Chemicals and reagents

All chemicals and reagents used for the quantification of metals and metalloids are of the highest purity and suitable for ICP-MS. Ultrapure water was obtained via a Milli-Q system (Millipore, Darmstadt, Germany). Hydrochloric acid 37% was purchased from Carlo Erba Reagents Srl (Arese, Italy). Nitric acid (65%) was obtained from Merck (Milan, Italy). Multi- and single-element standards, including CMS-2, CMS-3, CMS-4, CMS-5, MSHG-10PPM, and CGGD1, were purchased from Inorganic Ventures (Christiansburg, VA, United States). The Seronorm Trace Elements Urine L-1 (Product No. 210605) and L-2 (Product No. 210705), used as quality controls, were purchased from SERO (Billingstad, Norway).

### Inductively Coupled Plasma Mass Spectrometry (ICP-MS) analyses

Quantification of several metals and metalloids in CSF was performed by ICP-MS: calcium (Ca), copper (Cu), iron (Fe), strontium (Sr), manganese (Mn), molybdenum (Mo), beryllium (Be), cesium (Cs), barium (Ba), gadolinium (Gd), bismuth (Bi), antimony (Sb), mercury (Hg), cobalt (Co), chromium (Cr), lead (Pb), silver (Ag), nickel (Ni), zinc (Zn), arsenic (As), vanadium (V), and cadmium (Cd). A buffer solution (0.6% nitric acid and 0.6% hydrochloric acid) was prepared fresh before analysis for the dilution of calibrators, quality controls, and samples. Calibration curves (ranging from 1 to 100 ppb) were constructed through the analysis of multi- and single-element standards that had previously been diluted in buffer solution. Once thawed and gently mixed, CSF samples were diluted 20-fold in buffer. Likewise, to verify the accuracy of the data, certified quality controls (Seronorm Trace Elements Urine L-1 and L-2) were resuspended and diluted 20 times in buffer solution on the day of the analysis, and subsequently analyzed. Quantitative analyses were performed using an iCAP RQ ICP-MS (Thermo Scientific, Milan, Italy) equipped with an ASX-560 autosampler and sample introduction system (Thermo Scientific, Milan, Italy). Operation conditions were optimized daily to achieve maximum sensitivity, an oxide ratio <1% (140Ce16O+/140Ce+), and a doubly charged ratio <2% (140Ce2+/140Ce+). The instrument’s performance report was checked before each sample batch analysis to ensure proper instrument performance. To normalize analyte quantitative data, a Rh solution (10 ppb) was used as an internal standard, and its relative intensity was monitored in each sample. For each sample, metal and metalloid concentrations below measurable levels were assigned a value of 0.

### Bio-Plex Pro 37-plex inflammatory cytokine assays

CSF samples were thawed, briefly mixed, and centrifuged before cytokine quantification with the magnetic bead-based multiplex kit (37-plex Bio-Plex Pro™ Human Inflammation Panel 1, cod. 171AL001M, BioRad, United States), according to the manufacturer’s instructions. CSF samples were used undiluted. Quantification was performed with a Bio-Plex 200 system and Bio-Plex Manager™ 6.1 software (BioRad), using a five-parameter logistic regression formula to calculate sample concentrations from the standard curves. For each sample, cytokine concentrations below measurable levels were assigned a value of 0.

### Statistical analyses

Differences in patient age between the Control and Case Groups were assessed with the Mann-Whitney U test, while differences in sex, residence, and living environment were assessed with the Chi-squared test when all expected counts were five or more, or Fisher’s test when any expected count was less than five.

When comparing analyte levels across metals, metalloids, and cytokines, caution is warranted given the high proportion of undetectable samples. Undetectable values were assigned a value of zero for analysis, reflecting concentrations below the detection capability rather than true absence, and allowing consistent statistical comparisons across samples. In light of this, to evaluate differences in CSF samples between pediatric patient groups, two statistical methods were employed based on the detection frequency of each analyte. Metals and cytokines that were detectable in more than 30% of samples were analyzed as continuous variables. In this case, differences in concentrations were assessed using the Mann–Whitney U test. For each analyte, Benjamini–Hochberg false discovery rate (FDR) correction was applied to adjust p-values for multiple comparisons.

Metals, metalloids, and cytokines detectable in less than 30% of samples were analyzed as binary variables (measurable vs. non-measurable). For each analyte, detection frequencies were compared between groups. The Chi-squared test was used when all expected cell counts were five or more. When the expected count was less than five or the observed count was less than five, Fisher’s test was used. *P*-values were adjusted using the Benjamini–Hochberg FDR method.

To assess the correlation between CSF metals and metalloids and cytokines in pediatric patients, Spearman correlation analyses were performed. Only analytes with a detection rate of greater than 30% were included in this analysis. Correlation coefficients (r) were adjusted for multiple testing using the Benjamini–Hochberg FDR method.

Significant correlations with adjusted *p*-values <0.05 were highlighted, and their strengths and directions were visualized in a correlation matrix. The correlation matrix displayed only significant correlations using a color gradient, with correlation coefficients and significance indicated by text and asterisks (*: *p* < 0.05, **: *p* < 0.01, ***: *p* < 0.001).

The heatmaps were generated using row-wise Z-score normalization to emphasize relative concentration differences across all patients. Hierarchical clustering was performed on both metals and cytokines, as well as on patient samples, to detect patterns and subgroups. This integrative approach enabled intuitive visualization of group-dependent variations in metal concentrations across samples.

Multivariate analysis was performed to test the independence of the significant effects identified in univariate analyses. For these multivariate analyses, generalized linear models of the appropriate family were fitted, with the covariates significant in the univariate analyses included as independent variables. All statistics and graphs were obtained using the R Studio software (R version 4.5.1). Differences were considered significant when the *p-*value *<*0.05.

## Results

### Patient characteristics and underlying diseases

Patient characteristics are summarized in Table 1. We collected CSF from 69 (100%) children with hematological malignancies, 33 (37.5%) with CNS infections, 14 (15.9%) with neurodegenerative diseases, and 41 (46.6%) with neurodevelopmental disorders. The Control and Case groups exhibited comparable age and sex demographics, with no significant differences. Some differences were observed in patients’ places of residence (*p* = 1.87 × 10^−7^), as 52.1% of the Control Group resided in Italy, compared with 85.5% of the Case Group. On the other hand, no significant differences were observed in the living environments, with approximately half of the patients in each group residing in either an urban or rural environment.

**TABLE 1:** Patient characteristics and underlying diseases.

Variables	Control group (n = 69)	Case group (n = 88)	*p*-value
Age, years, median (IQR)	8.11 (4.76–11.52)	9.89 (5.48–13.83)	^§^ 0.25
Sex, number (%) - Male - Female	47 (68.1%) 22 (31.9%)	45 (51.1%) 43 (48.9%)	0.071
Diagnosis, number (%) - Hematological malignancies - CNS infection - Neurodegenerative disease - Neurodevelopmental disorders	69 (100.0%) – – –	– 33 (37.5%) 14 (15.9%) 41 (46.6%)	NA
Patient’s residence at first symptoms, number (%) - Northern Italy - Southern Italy - Albania - Romania	31 (44.9%) 5 (7.2%) 26 (37.7%) 7 (10.1%)	56 (63.6%) 21 (23.9%) 3 (3.4%) 8 (9.1%)	^†^ 1.87x10^−7^
Patient’s living environment at first symptoms, number (%) - Urban - Rural	32 (46.4%) 37 (53.6%)	49 (55.7%) 39 (44.3%)	0.32

P-values in the respective column refer to Chi-squared tests, unless indicated otherwise: § Mann-Whitney U test, † Fisher test. Abbreviations: IQR, interquartile range; NA, not applicable.

### Patients with CNS diseases exhibit higher concentrations of metals and metalloids in their CSF

ICP-MS analyses were performed to measure the concentration of the following 22 metals and metalloids in the CSF of Case and Control Groups: calcium (Ca), copper (Cu), iron (Fe), strontium (Sr), manganese (Mn), molybdenum (Mo), beryllium (Be), cesium (Cs), barium (Ba), gadolinium (Gd), bismuth (Bi), antimony (Sb), mercury (Hg), cobalt (Co), chromium (Cr), lead (Pb), silver (Ag), nickel (Ni), zinc (Zn), arsenic (As), vanadium (V), cadmium (Cd).

**FIGURE 1:**
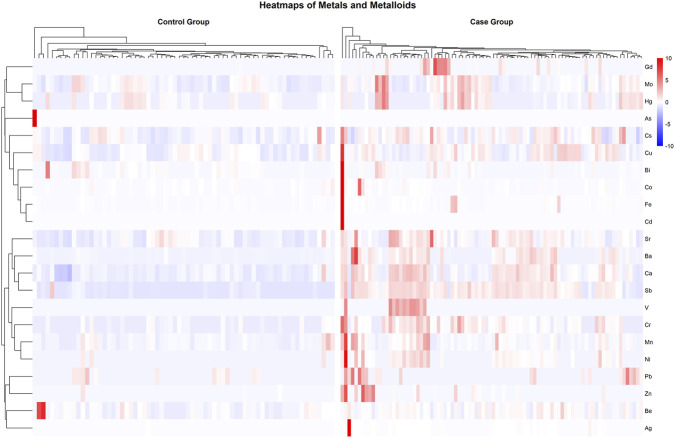
Heatmaps showing the relative concentrations of metals and metalloids in CSF samples. Hierarchical clustering was performed both on the metals/metalloids (y-axis) and the patient samples (x-axis), revealing patterns of accumulation within patient groups. Color intensity represents the relative concentration of each metal/metalloid, expressed as row-wise Z-score, with blue indicating lower concentrations and red indicating higher concentrations.

To obtain an overview of the variability among patients in metal and metalloid concentrations, hierarchical clustering was performed on both metals and patient samples (Figure 1). Stratification of the Case Group by disease type was not performed, as no meaningful differences in metal and metalloid profiles were observed between patients with CNS infection and those with other neurological diseases, and did not contribute to further interpretation of the data. The results shown in the heatmap suggest higher concentrations of metals and metalloids in Case patients compared to the Controls. Additionally, some metals and metalloids tend to cluster together, indicating similar profiles: the most striking example is represented by Mo and Hg, which exhibit similar behavior among patients. In addition, Sr, Ba, Ca, and Sb also show fairly similar profiles within the Case Group. The full data set detailing the concentration and detectability values of all metals and metalloids is provided in Supplementary Table S1. Data show Ca as the most abundant essential element in CSF (medians of 6842.68 ng/mL in Control Group and 9139.65 ng/mL in Case Group; adj *p*-value: 3.67 × 10^−12^), followed by Cu (129.78 ng/mL and 166.10 ng/mL; adj *p*-value: 8.58 × 10^−5^) and Fe (19.43 ng/mL and 41.99 ng/mL; adj *p*-value: 1.83 × 10^−10^).

**FIGURE 2:**
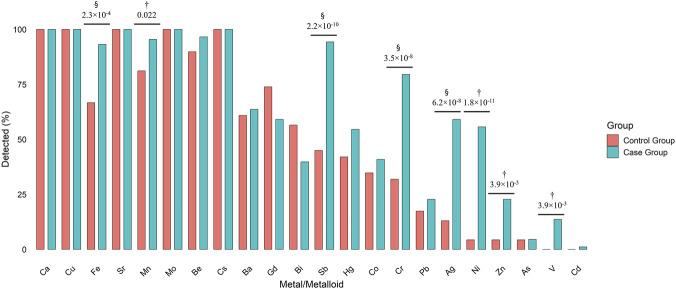
Grouped bar graph showing the percentage of cerebrospinal fluid (CSF) samples in which each metal or metalloid was detected in the case and control groups. Bars are colored by sample group: red = Control Group, blue = Case Group. Metals and metalloids are shown on the X-axis, and detection frequency on the Y-axis. p-values were calculated using Fisher’s exact test or Chi squared test and adjusted using Benjamini–Hochberg FDR. Adjusted p-values are shown only for metals and metalloids that showed significant differences between patient groups. § Chi-squared test, † Fisher test.

The majority of metals and metalloids were detected only at trace levels, with some detectable in the majority of CSF samples (100% for Ca, Cu, Sr, Figure 2). In comparison, others were quantifiable in a few or no samples (4%–5% for As, 0%–14% for V, and 0%–1% for Cd, Figure 2). A comparison of the two patient groups shows that many elements are more frequently detected in the Case Group compared to the Controls, suggesting that the concentrations of these analytes are higher in the CSF of the Case Group. For instance, the detectability of Fe increases from 67% in Controls to 93% in the Case Group (Figure 2). Other notable examples of this phenomenon are Sb (45% vs 94%), Cr (32% vs 80%), Ag (13% vs 59%), Ni (4% vs 56%), and Zn (4% vs 23%) (see Supplementary Table S1; Figure 2).

**FIGURE 3:**
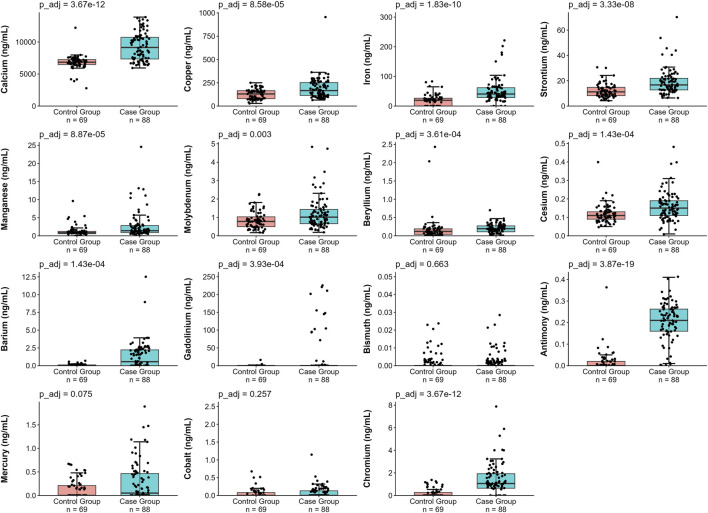
Boxplots showing cerebrospinal fluid (CSF) concentrations of selected metals and metalloids in the case and control groups. Individual data points are depicted as black dots. Boxplots represent the interquartile range (IQR) with median values indicated, and whiskers extend to 1.5× IQR. The y-axis was custom-scaled for Iron (0–300 ng/mL), Gadolinium (0–250 ng/mL), Bismuth (0–0.05 ng/mL), and Cobalt (0–2.5 ng/mL) to improve visualization of the sample distributions. Adjusted *p*-values (*p*_adj, Benjamini–Hochberg correction) comparing the two groups are displayed on top of each plot. Sample sizes for each group are indicated below each plot.

As illustrated in Figure 3, the concentrations of metals and metalloids were compared between the two groups. For the metals and metalloids detected in more than 30% of the samples (i.e., from Ca to Cr in Supplementary Table S1; Figure 2), we generally observed higher levels in the Case Group patients, except Bi, Hg, and Co, which showed no significant differences. Analysis of the metals and metalloids detected in less than 30% of the samples (i.e., from Pb to Cd) highlighted a significant increase in the ability to measure Ag, Ni, Zn, and V in the Case Group. The detectability of Pb did not differ between groups, whereas As and Cd were detectable in only a few samples.

To understand which metals and metalloids are significantly associated with the Case Group, a multivariate logistic regression analysis was conducted. A significant association was identified between the Case Group and the following elements: Hg (OR = 1.60 × 10^4^), Sb (OR = 2.07 × 10^9^), Pb (OR = 4.08 × 10^17^), and Cu (OR = 1.018).

### Control and Case Group patients display different CSF cytokine profiles

**FIGURE 4:**
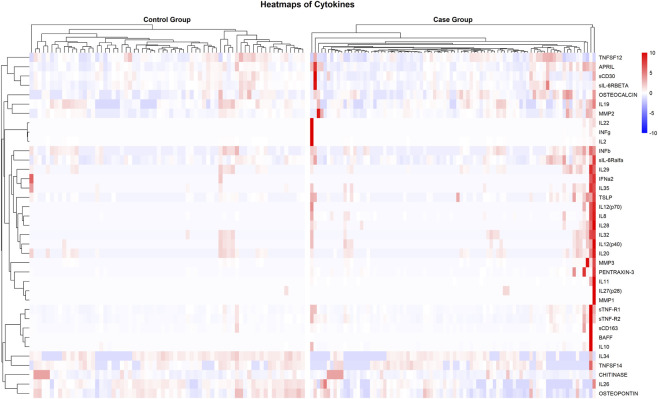
Heatmaps showing the relative concentrations of inflammatory cytokines in cerebrospinal fluid (CSF) samples. Hierarchical clustering was performed both on the cytokines (y-axis) and the patient samples (x-axis), revealing patterns of accumulation within patient groups. Color intensity represents the relative concentration of each cytokine, expressed as row-wise Z-score, with blue indicating lower concentrations and red indicating higher concentrations.

To account for the inflammatory state of the CNS in each patient, a panel of 37 human inflammation-related analytes in CSF was measured using Bio-Plex Pro 37-Plex assays. A hierarchical clustering analysis was performed to visually summarize the variability in cytokine profiles across the patient cohort (Figure 4). The resulting heat map shows substantial heterogeneity in cytokine levels across all patients, regardless of assigned study group.

The full dataset detailing the concentration and detectability values for all cytokines is provided in Supplementary Table S2.

**FIGURE 5:**
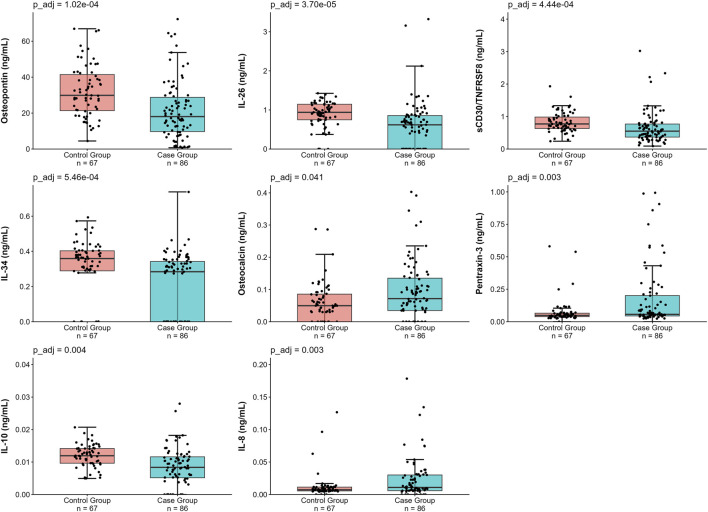
Boxplots showing cerebrospinal fluid (CSF) concentrations of the cytokines with significant differences in the case and control groups. Individual data points are depicted as black dots. Boxplots represent the interquartile range (IQR) with median values indicated, and whiskers extend to 1.5× IQR. The y-axis was custom-scaled for sCD30/TNFRSF8 (0–4 ng/mL), Pentraxin-3 (0–1 ng/mL), IL-10 (0–0.04 ng/mL), and IL-8 (0–0.2 ng/mL) to improve visualization of the sample distributions. Adjusted *p*-values (*p*_adj, Benjamini–Hochberg correction) comparing the two groups are displayed on top of each plot. Sample sizes for each group are indicated below each plot.

The following cytokines showed significantly higher levels in the Control Group compared to the Case Group: Osteopontin (29.14 ng/mL vs 18.04 ng/mL; adj *p*-value: 1.02 × 10^−4^), IL-26 (0.92 ng/mL vs 0.62 ng/mL; adj *p*-value: 3.7 × 10^−5^), sCD30/TNFRSF8 (0.77 ng/mL vs 0.56 ng/mL; adj *p*-value: 4.44 × 10^−4^), IL-34 (0.36 ng/mL vs 0.28 ng/mL; adj *p*-value: 5.46 × 10^−4^) and IL-10 (0.012 ng/mL vs 0.092 ng/mL; adj *p*-value: 0.004) (Supplementary Table S2; Figure 5). On the other hand, three cytokines showed a significantly higher concentration in the Case Group: Osteocalcin (0.050 ng/mL vs 0.072 ng/mL; adj *p*-value: 0.041), Pentraxin-3 (0.048 ng/mL vs 0.066 ng/mL; adj *p*-value: 0.003) and IL-8 (0.0073 ng/mL vs 0.013 ng/mL; adj *p*-value: 0.003) (Supplementary Table S2). Lastly, IL-28A/IFN-gamma2 showed higher detectability in the Case Group (0% vs 12%; adj *p*-value: 0.041).

A multivariate logistic regression analysis was performed to identify cytokines associated with either patient group. However, the odds ratios for all cytokines were not significantly different from 1, indicating that these molecules are not reliable predictors of patient group membership.

### Correlation analyses between metals and metalloids versus inflammatory cytokines

After identifying the metals, metalloids, and cytokines that differed significantly between the two patient groups, we performed correlation analyses to determine whether these variables showed similar behavior in CSF samples. Only the metals, metalloids, and cytokines that had more than 30% detectable samples were included in this analysis.

**FIGURE 6:**
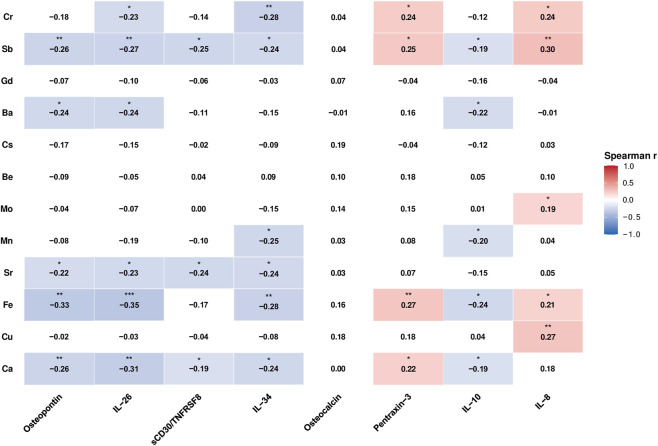
Heatmap showing Spearman correlation coefficients between cerebrospinal fluid (CSF) concentrations of metals and metalloids (y-axis) and cytokines (x-axis). Only statistically significant correlations after FDR correction (adj *p*-value: <0.05) are color-coded, with blue representing negative correlations and red representing positive correlations. All correlation coefficients are displayed within the cells, and significance levels are indicated by stars (* adj *p*-value: <0.05, ** adj *p*-value: <0.01, *** adj *p*-value: <0.001). Non-significant correlations are shown in white.

Pentraxin-3 and IL-8 both showed a weak positive correlation with Fe, Sb, and Cr (Figure 6). Pentraxin-3 also displayed a weak correlation with Ca, while IL-8 showed a weak correlation with Cu. Instead, Osteocalcin showed no significant correlation with any metals.

On the other hand, Osteopontin, IL-26, sCD30/TNFRSF8, IL-34, and IL-10 showed weak negative correlations with Ca, Fe, Sr, Mn, Ba, Sb, and Cr (see Figure 6).

## Discussion

In this study, we aimed to characterize metal and metalloid concentrations in the CSF of pediatric patients and to examine their relationship with neuroinflammation. To this end, we quantified metal and metalloid concentrations together with inflammatory cytokine markers in the CSF of children with neurological disease or CNS infection. Our results reveal altered CSF metal and metalloid profiles in these patients, accompanied by distinct inflammatory cytokine patterns, supporting a potential link between metal dysregulation and neuroinflammation.

A key finding is the overall increase in the concentration and detection rates of most metals and metalloids in the Case Group (neurological/infection patients), indicating significant CNS dysregulation. These elements may be grouped by biological relevance and potential toxicity. For instance, essential trace elements such as Ca, Sr, Cu, Fe, Mn, Zn, Co, and Mo are required for normal physiological functions. However, they may display toxicity at high concentrations (Jomova et al., 2022). Interestingly, we observed higher concentrations of all these essential elements in the Case Group, except for Zn, for which only an increased detection frequency was observed.

Other metals are generally considered toxic or harmful, including Cr, Sb, Pb, Hg, Cd, and As (Peana et al., 2021). In this case, only Sb and Cr showed significantly higher concentrations in the Case Group, whereas Hg and Pb showed no significant difference between the patient groups. Regarding As and Cd, their levels were too low to be consistently detected.

Lastly, Bi, V, Cs, Be, Ba, Ag, Ni, and Gd have limited or no biological function, and their accumulation is likewise seen in neurological and infected patients.

The buildup of essential metals could reflect enhanced molecular signaling and metabolic activity or a malfunction in cellular repair mechanisms within the affected CNS, often linked to increased oxidative stress and inflammation (Chen et al., 2016; Mezzaroba et al., 2019).

In fact, several mechanisms contribute to metal-induced neurotoxicity. Metals such as Pb, Hg, Cd, and Mn generate reactive oxygen species and impair mitochondrial function, leading to oxidative stress and cellular injury (Ijomone et al., 2025). In neurons, these mechanisms can disrupt synaptic function by interfering with neurotransmitter receptors, cytoskeletal proteins, and intracellular signaling pathways, compromising synaptic plasticity and neuronal connectivity (Carmona et al., 2021). Moreover, metal exposure activates microglia and astrocytes, promoting chronic neuroinflammation and pro-inflammatory cytokine release, which further exacerbates neuronal dysfunction (Martínez-Hernández et al., 2023; Wei et al., 2024). Finally, metals can impair blood–brain barrier integrity, facilitating CNS accumulation of potentially toxic elements and amplifying neurotoxicity (Zahoor et al., 2024).

Although Ca, Sr, Cu, Fe, Mn, Zn, Co, and Mo are essential for normal cellular and neuronal function, disruption of their homeostasis may lead to significant neurotoxic consequences, through the aforementioned mechanisms. Furthermore, the toxic metals Sb and Cr, of which we observe an increase in patients with neurological diseases or CNS infections, also exert deleterious effects on the nervous system through similar mechanisms. In fact, Sb exposure was observed to induce autophagy, leading to neuronal cell death in mice models in a dose-dependent manner (Wang et al., 2019). Zebrafish embryos also showed sensitivity to Sb exposure, with neurotoxic effects leading to developmental abnormalities and behavior defects, also accompanied by increased oxidative stress (Xia et al., 2021). Another study in Zebrafish showed diminished integrity of the BBB following Sb exposure, with a breakdown of the basal lamina, tight junctions and nerve fibers in the brain (Xu et al., 2024).

Cr exposure has likewise been linked to mitochondrial dysfunction and oxidative stress in neurons, in addition to alteration of neurodevelopment-related gene expression and epigenetic profiles (Xu et al., 2021; Zhang et al., 2024).

Accumulation of toxic and non-biologically relevant metals may be linked to both environmental exposure and increased CNS barrier permeability. For instance, substantial evidence indicates that CNS pathologies are characterized by BBB dysfunction (Abbott et al., 2010), which may facilitate the accumulation of metals/metalloids in the CSF, thereby further promoting oxidative stress and neuroinflammation.

Regarding cytokine profiles, Osteopontin, IL-26, IL-34, and IL-10 were elevated in the CSF of the Control Group, suggesting a distinct profile in this population. In terms of their functions, studies indicate that Osteopontin is increased in the CSF of Alzheimer’s disease and multiple sclerosis patients (Comi et al., 2010; Orsi et al., 2021; Marastoni et al., 2024), supporting a role in neurological pathologies, and there is emerging evidence for its involvement in pediatric cancers, although further studies are needed to clarify this association (Karpinsky et al., 2017). IL-26, while generally considered proinflammatory (Gowhari Shabgah et al., 2022), has also been linked to reduced blood-brain barrier permeability and attenuated severity of autoimmune encephalomyelitis in mice (Broux et al., 2020), indicating potential protective or regulatory roles in the CNS. IL-34 contributes to microglial survival and the maintenance of immune homeostasis in the CNS, but its role in neurological conditions remains poorly understood (Chhetri, 2023). Finally, IL-10 has been used as a biomarker to distinguish between conditions such as Multiple Sclerosis, characterized by lower levels, and Neuro-Behçet’s Disease, characterized by higher levels (Belghith et al., 2018). Additionally, it has proven useful as a biomarker in primary and secondary CNS lymphoma (Nguyen-Them et al., 2016; Gu et al., 2024). Conversely, elevated levels of Osteocalcin, Pentraxin-3, and IL-8 were observed in patients with neurological conditions and CNS infections. Although Osteocalcin has been associated with beneficial effects on brain function (Shan et al., 2019; Qi et al., 2024), it is also linked to the pathogenesis of Alzheimer’s disease (Bu et al., 2025), reflecting a multifaceted role in the CNS. Pentraxin-3 levels are generally increased during CNS infections (Rajkovic et al., 2019; Zatta et al., 2020; Thomsen et al., 2023; El-Kady et al., 2025), consistent with its role as an inflammatory mediator. IL-8 is associated with increased blood-brain barrier permeability (Kossmann et al., 1997) and has been proposed as a biomarker for sepsis-associated encephalopathy (Mao et al., 2023) and other CNS diseases (Kmezic et al., 2023), reflecting its involvement in acute neuroinflammatory processes (Asano et al., 2010).

From our study, correlation analyses suggest that Pentraxin-3 positively correlates with Ca, Fe, Sb, and Cr, and IL-8 positively correlates with Cu, Fe, Mo, Sb, and Cr in the CSF of patients. This finding establishes a molecular link, hypothesizing that excess metal acts as a critical stressor that triggers Pentraxin-3- and IL-8-mediated neuroinflammatory pathways, thereby contributing to neurological pathology. However, further research is needed to determine whether these relationships are functionally meaningful. To better understand the interplay between specific cytokine profiles and metals and metalloids, longitudinal studies are required to reveal how both sets of variables change during disease progression or patient treatment and to clarify how these dynamics relate to disease state.

A limitation of this study is the use of oncological patients at diagnosis as the Control Group, as CSF collection from healthy children is ethically unfeasible. Although no malignant involvement of the CNS was observed, thereby minimizing the potential impact on their CSF profiles, these patients may exhibit elevated systemic levels of inflammatory molecules, which could be reflected in the CNS. Metabolic dysfunctions or pharmacological treatments may also lead to abnormal metal accumulation, triggering oxidative stress, microglial activation, and neuroinflammation, which, in turn, promote disease progression by increasing BBB permeability (Bañuelos-Cabrera et al., 2014; Kim et al., 2025).

Another limitation is that no information was collected on patients' lifestyles or potential environmental exposure to metals and metalloids, which prevented us from considering these factors. Nonetheless, it should be noted that all patients included in this study reside in geographically close countries that share similar environmental and industrial conditions and have not experienced recent ecological disasters. As a result, differences in country of residence between patient groups are unlikely to affect CSF levels of environmentally relevant metals and metalloids.

Lastly, it is difficult to assess the magnitude of metal accumulation in the CSF of our patients, as no reference values are available for healthy pediatric controls. A comparison with studies measuring the levels of these elements in the biological fluids of healthy adult controls may be performed. However, we observe variability in metal concentrations between our study and others (Goullé et al., 2005; Bocca et al., 2010; Zhai et al., 2017; Stojsavljević et al., 2020), likely due to differences in patient populations and methodological variations. These inconsistencies solidify the need for longitudinal studies to more accurately characterize metal accumulation over time and clarify its relationship to disease state.

In conclusion, a general increase in metals and metalloids in the CSF of children with neurological diseases and infections was observed compared to our control group. Distinct cytokine profiles were also observed, with modest correlations between Pentraxin-3 and IL-8 levels and the accumulation of Ca, Cu, Fe, Mo, Sb, and Cr. Together, these findings point to alterations in metal homeostasis associated with CNS pathology, potentially reflecting shared biological mechanisms across neurological disorders despite differences in etiology and pathophysiology.

Several processes may contribute to metal accumulation in the CNS, including neuroinflammation, blood–brain barrier dysfunction, and impaired metal transport and clearance. The observed correlations between metals and inflammatory cytokines, while modest in strength, suggest a complex interplay between metal homeostasis and neuroinflammatory processes, reflecting biologically relevant pathophysiological mechanisms.

Overall, this work provides a comprehensive assessment of CSF metal and metalloid profiles in pediatric patients, integrated with inflammatory markers. While further validation is required, these findings lay the groundwork for future longitudinal studies aimed at clarifying the role of metal–neuroinflammation interactions in disease activity, progression, and therapeutic response.

## Data Availability

The original contributions presented in the study are included in the article/Supplementary Material, further inquiries can be directed to the corresponding author.
