# Supplementary material for: Comparative analysis reveals distinct metal and inflammatory cytokine profiles in the cerebrospinal fluid of children with neurological disorders
Source: Front Toxicol. 2026 Mar 24;8:1766904. doi: 10.3389/ftox.2026.1766904 (PMC13053030; doi:10.3389/ftox.2026.1766904)
Supplement: Supplementary file 1 [file Table1.docx]

**Table S1: Concentration and detectability of metals/metalloids in cerebrospinal fluid samples of pediatric patients.**

|  | **Control group** | | **Case group** | |  |
| --- | --- | --- | --- | --- | --- |
| **Element** | **Detected (%)** | **Median (Q1, Q3)** | **Detected (%)** | **Median (Q1, Q3)** | **adj p-value** |
| Ca | 100% | 6842.68 (6475.98, 7247.26) | 100% | 9139.65 (7348.65, 10728.57) | 3.67 × 10^-12^ |
| Cu | 100% | 129.78 (79.75, 165.34) | 100% | 166.1 (114.05, 251.96) | 8.58 × 10^-5^ |
| Fe | 67% | 19.43 (0, 26.5) | 93% | 41.99 (30.67, 64.75) | 1.83 × 10^-10^ |
| Sr | 100% | 11.2 (8.19, 14.76) | 100% | 16.69 (12.74, 21.92) | 3.33 × 10^-8^ |
| Mn | 81% | 0.87 (0.59, 1.23) | 95% | 1.41 (0.9, 2.83) | 8.87 × 10^-5^ |
| Mo | 100% | 0.78 (0.5, 1.03) | 100% | 1.01 (0.66, 1.43) | 3.07 × 10^-3^ |
| Be | 90% | 0.12 (0.04, 0.19) | 97% | 0.19 (0.11, 0.28) | 3.61 × 10^-4^ |
| Cs | 100% | 0.11 (0.09, 0.13) | 100% | 0.15 (0.11, 0.19) | 1.43 × 10^-4^ |
| Ba | 61% | 0.04 (0, 0.11) | 64% | 0.56 (0, 2.23) | 1.43 × 10^-4^ |
| Gd | 74% | 0.002 (0, 0.003) | 59% | 0.0008 (0, 2.73) | 3.93 × 10^-4^ |
| Bi | 57% | 0.0003 (0, 0.004) | 40% | 0 (0, 0.002) | 0.66 |
| Sb | 45% | 0 (0, 0.02) | 94% | 0.21 (0.16, 0.26) | 3.87 × 10^-19^ |
| Hg | 42% | 0 (0, 0.21) | 55% | 0.05 (0, 0.47) | 0.074 |
| Co | 35% | 0 (0, 0.08) | 41% | 0 (0, 0.14) | 0.26 |
| Cr | 32% | 0 (0, 0.26) | 80% | 1.06 (0.64, 1.93) | 3.67 × 10^-12^ |
| Pb | 17% | 0 (0, 0) | 23% | 0 (0, 0) | ^§^ 0.84 |
| Ag | 13% | 0 (0, 0) | 59% | 0.02 (0, 0.05) | ^§^ 6.21 × 10^-8^ |
| Ni | 4% | 0 (0, 0) | 56% | 1.5 (0, 4.09) | ^†^ 1.79 × 10^-11^ |
| Zn | 4% | 0 (0, 0) | 23% | 0 (0, 0) | ^†^ 0.0039 |
| As | 4% | 0 (0, 0) | 5% | 0 (0, 0) | ^†^ 1 |
| V | 0% | 0 (0, 0) | 14% | 0 (0, 0) | ^†^ 0.0039 |
| Cd | 0% | 0 (0, 0) | 1% | 0 (0, 0) | ^†^ 1 |

Metals/metalloids are ordered according to their median levels (most abundant to least abundant). The Detected (%) columns display the number of samples in which the metal could be measured. Medians, along with the first and third quartiles, are provided in ng/mL. Adjusted p-values for metals/metalloids with more than 30% detectable samples (above red line) were calculated using Mann–Whitney U tests and adjusted for multiple comparisons using Benjamini–Hochberg FDR. Adjusted p-values for metals/metalloids with less than 30% detectable samples (below red line) were calculated using Fisher’s exact test or Chi squared test and adjusted using Benjamini–Hochberg FDR. Adjusted p-values in the respective column refer to Mann-Whitney tests, unless indicated otherwise: § Chi-squared test, † Fisher test. Abbreviations: Ca, calcium; Cu, copper; Fe, iron; Sr, strontium; Mn, manganese; Mo, molybdenum; Be, beryllium; Cs, cesium; Ba, barium; Gd, gadolinium; Bi, bismuth; Sb, antimony; Hg, mercury, Co, cobalt; Cr, chromium, Pb, lead; Ag, silver; Ni, Nickel; Zn, zinc; As, arsenicum; V, vanadium; Cd, cadmium.
