# Supplementary material for: Comparative analysis reveals distinct metal and inflammatory cytokine profiles in the cerebrospinal fluid of children with neurological disorders
Source: Front Toxicol. 2026 Mar 24;8:1766904. doi: 10.3389/ftox.2026.1766904 (PMC13053030; doi:10.3389/ftox.2026.1766904)
Supplement: Supplementary file 2 [file Table2.docx]

**Table S2: Concentration and detectability of cytokines in cerebrospinal fluid samples of pediatric patients.**

|  | **Control group** | | | **Case group** | |  |
| --- | --- | --- | --- | --- | --- | --- |
| **Cytokine** | **Detected (%)** | **Median (Q1, Q3)** | **Detected (%)** | | **Median (Q1, Q3)** | **adj p-value** |
| gp130/sIL-6RBETA | 100% | 32.06 (22.90, 38.18) | 100% | | 25.38 (18.85, 33.40) | 0.053 |
| OSTEOPONTIN | 100% | 29.14 (21.20, 40.21) | 100% | | 18.05 (9.72, 28.78) | 1.02 × 10^-4^ |
| APRIL/TNFSF | 100% | 19.62 (13.93, 25.68) | 99% | | 17.45 (12.02, 26.27) | 0.82 |
| CHITINASE 3-LIKE 1 | 100% | 9.78 (7.75, 13.60) | 100% | | 9.10 (7.64, 12.68) | 0.60 |
| sCD163 | 100% | 6.76 (4.81, 9.59) | 100% | | 6.49 (4.57, 14.93) | 0.69 |
| BAFF/TNFSF13B | 100% | 5.69 (4.66, 7.93) | 100% | | 5.88 (4.39, 10.00) | 0.55 |
| sTNF-R1 | 100% | 1.49 (1.12, 1.88) | 100% | | 1.64 (1.08, 2.55) | 0.35 |
| sIL-6Ralfa | 100% | 1.15 (0.81, 1.59) | 100% | | 1.14 (0.78, 1.74) | 0.93 |
| IL-26 | 93% | 0.92 (0.75, 1.13) | 67% | | 0.62 (0, 0.86) | 3.7 × 10^-5^ |
| sCD30/TNFRSF8 | 100% | 0.77 (0.64, 0.98) | 100% | | 0.56 (0.37, 0.78) | 4.44 × 10^-4^ |
| TWEAK/TNFSF12 | 100% | 0.49 (0.40, 0.59) | 100% | | 0.44 (0.30, 0.60) | 0.32 |
| MMP-2 | 79% | 0.36 (0.20, 0.64) | 81% | | 0.44 (0.23, 0.66) | 0.35 |
| IL-34 | 80% | 0.36 (0.29, 0.40) | 56% | | 0.28 (0, 0.34) | 5.46 × 10^-4^ |
| sTNF-R2 | 100% | 0.098 (0.090, 0.11) | 100% | | 0.10 (0.086, 0.15) | 0.60 |
| OSTEOCALCIN | 74% | 0.050 (0.007, 0.090) | 79% | | 0.072 (0.035, 0.13) | 0.041 |
| PENTRAXIN-3 | 97% | 0.048 (0.039, 0.066) | 97% | | 0.066 (0.046, 0.25) | 0.003 |
| IL-19 | 83% | 0.041 (0.031, 0.049) | 80% | | 0.038 (0.027, 0.049) | 0.60 |
| IL-10 | 99% | 0.012 (0.0096, 0.014) | 85% | | 0.092 (0.0052, 0.0124) | 0.004 |
| LIGHT/TNFSF14 | 77% | 0.0087 (0.0064, 0.012) | 60% | | 0.0093 (0, 0.012) | 0.72 |
| IL-8 | 90% | 0.0073 (0.0055, 0.011) | 86% | | 0.013 (0.0064, 0.038) | 0.003 |
| INF-BETA | 47% | 0 (0, 0.0012) | 44% | | 0 (0, 0.0020) | 0.79 |
| TSLP | 39% | 0 (0, 0.0014) | 42% | | 0 (0, 0.0018) | 0.42 |
| IL-2 | 21% | 0 (0, 0) | 27% | | 0 (0, 0.0016) | ^§^ 1 |
| IL-11 | 21% | 0 (0, 0) | 36% | | 0 (0, 0.56x10^-3^) | ^§^ 0.36 |
| IL-20 | 21% | 0 (0, 0) | 27% | | 0 (0, 0.026) | ^§^ 1 |
| IL-29/IFNgamma1 | 16% | 0 (0, 0) | 24% | | 0 (0, 0) | ^§^ 0.89 |
| MMP-3 | 16% | 0 (0, 0) | 23% | | 0 (0, 0) | ^§^ 1 |
| IL-12(p40) | 7% | 0 (0, 0) | 14% | | 0 (0, 0) | ^§^ 0.89 |
| IL-32 | 7% | 0 (0, 0) | 15% | | 0 (0, 0) | ^§^ 0.84 |
| IL-35 | 7% | 0 (0, 0) | 14% | | 0 (0, 0) | ^§^ 0.89 |
| IFN-alfa2 | 3% | 0 (0, 0) | 2% | | 0 (0, 0) | ^†^ 1 |
| IL-27(p28) | 1% | 0 (0, 0) | 3% | | 0 (0, 0) | ^†^ 1 |
| INF-gamma | 0% | 0 (0, 0) | 6% | | 0 (0, 0) | ^†^ 0.36 |
| IL-12(p70) | 0% | 0 (0, 0) | 5% | | 0 (0, 0) | ^†^ 0.54 |
| IL-22 | 0% | 0 (0, 0) | 5% | | 0 (0, 0) | ^†^ 0.54 |
| IL-28A/IFN-gamma2 | 0% | 0 (0, 0) | 12% | | 0 (0, 0) | ^†^ 0.041 |
| MMP-1 | 0% | 0 (0, 0) | 1% | | 0 (0, 0) | ^†^ 1 |

Cytokines are ordered by median level (most abundant to least abundant). The Detected (%) column displays the number of samples in which the cytokine could be measured. Medians are provided, along with the first and third quartile, expressed in ng/mL. Adjusted p-values for cytokines with more than 30% detectable samples (above red line) were calculated using Mann–Whitney U tests and adjusted for multiple comparisons using Benjamini–Hochberg FDR. Adjusted p-values for cytokines with less than 30% detectable samples (below red line) were calculated using Fisher’s exact test or Chi squared test and adjusted using Benjamini–Hochberg FDR. Adjusted p-values in the respective column refer to Mann-Whitney tests, unless indicated otherwise: § Chi-squared test, † Fisher test.
